# Supplementary material for: Theoretical Investigation on the Hydrogen Evolution, Oxygen Evolution, and Oxygen Reduction Reactions Performances of Two-Dimensional Metal-Organic Frameworks Fe3(C2X)12 (X = NH, O, S)
Source: Molecules. 2022 Feb 24;27(5):1528. doi: 10.3390/molecules27051528 (PMC8912093; doi:10.3390/molecules27051528)
Supplement: Supplementary file 1 [file molecules-27-01528-s001.zip › molecules-1598121-supplementary.pdf]

Supporting Information

Theoretical Investigation on the Hydrogen Evolution, Oxygen Evolution, and Oxygen Reduction Reactions Performances of Two-Dimensional Metal-Organic Frameworks $Fe_3(C_2X)_{12}$ (X=NH, O, S)

Xiaohang Yang [1], Zhen Feng [2,3,*] and Zhanyong Guo [2]

1 School of Science, Henan Institute of Technology, Xinxiang 453000, China; yangxh@hait.edu.cn

2 School of Materials Science and Engineering, Henan Institute of Technology, Xinxiang 453000, China; guozhanyong123@126.com

3 School of Physics, Henan Normal University, Xinxiang 453007, China

* Correspondence: fengzhen@hait.edu.cn

Supplementary Figures

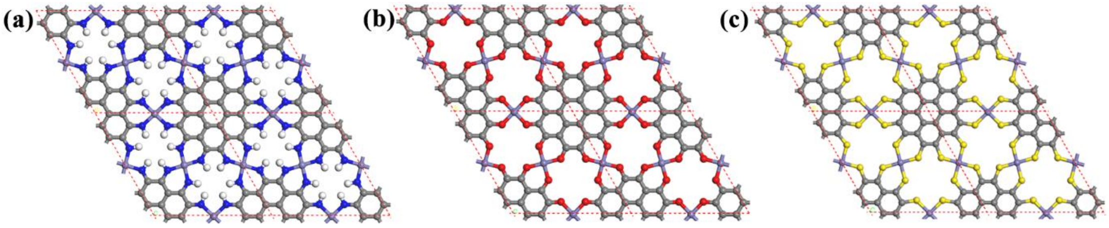

Figure S1. Supercell (2×2) of (a) Fe-NH-MOF, (b) Fe-O-MOF, and (c) Fe-S-MOF monolayers.

Materials	Adsorbate/ Site	Top view	Side view
Fe-NH-MOF	-	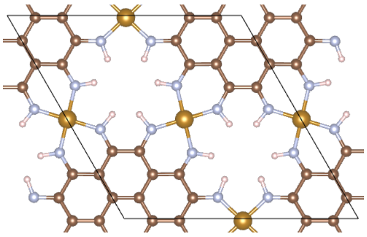	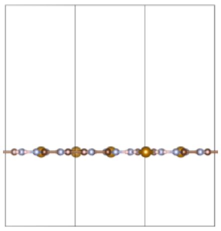
Fe-NH-MOF	H/ Fe	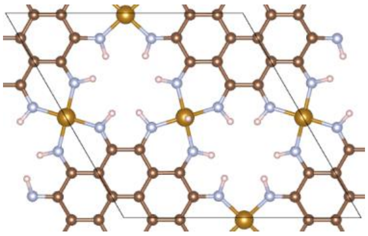	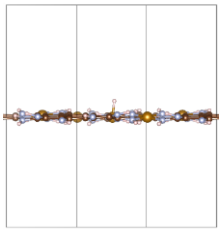
Fe-NH-MOF	H/ N	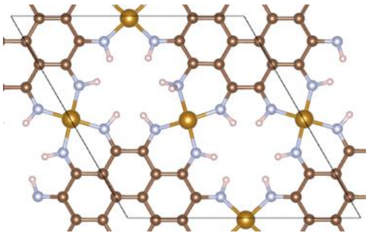	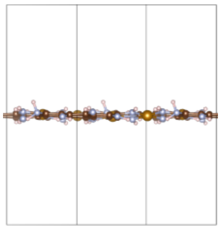
Fe-NH-MOF	H/C1	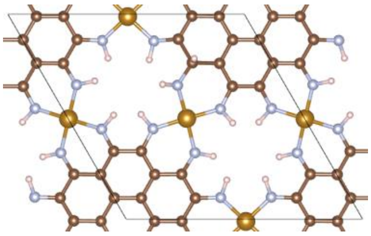	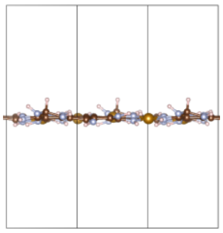
Fe-NH-MOF	H/C2	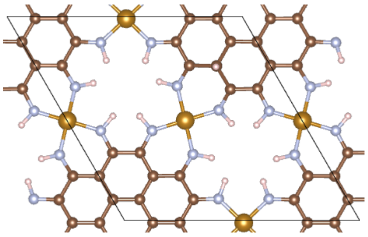	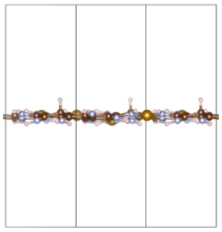
Fe-NH-MOF	H/C3	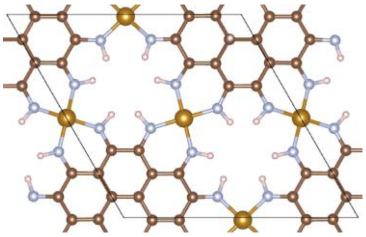	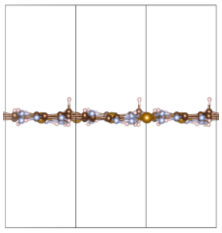

Fe-NH-MOF	OOH/Fe	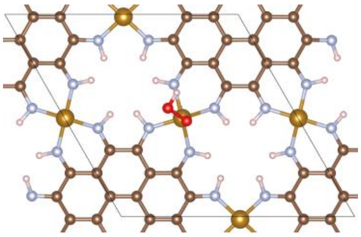	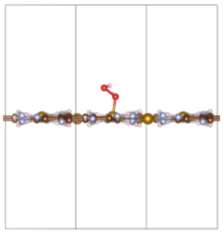
Fe-NH-MOF	OOH/N	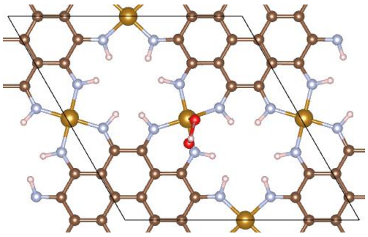	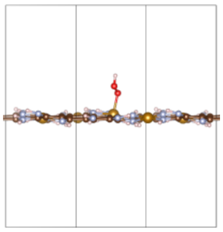
Fe-NH-MOF	OOH/C1	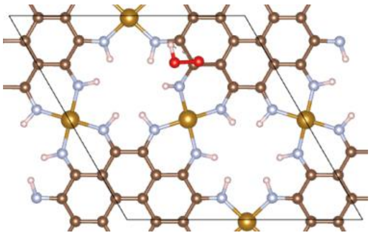	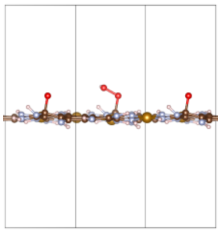
Fe-NH-MOF	OOH/C2	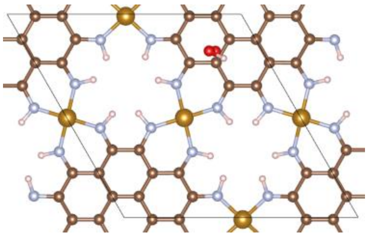	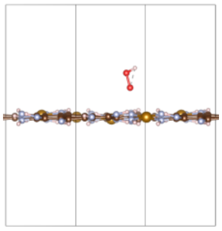
Fe-NH-MOF	OOH/C3	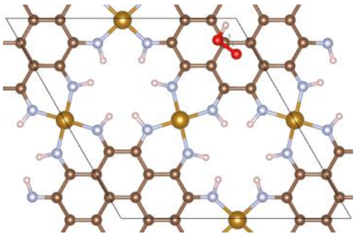	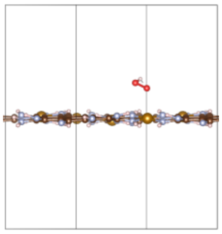
Fe-NH-MOF	OH/Fe	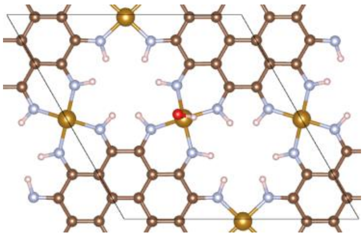	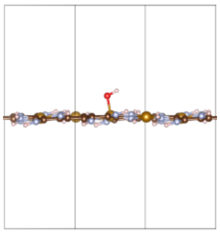
Fe-NH-MOF	OH/N	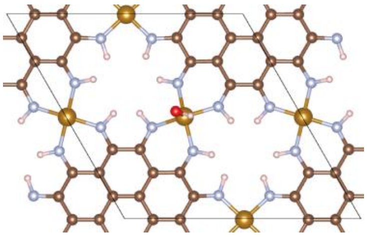	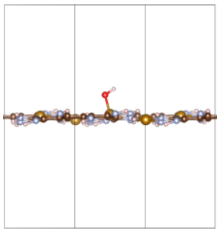

Fe-NH-MOF	OH/C1	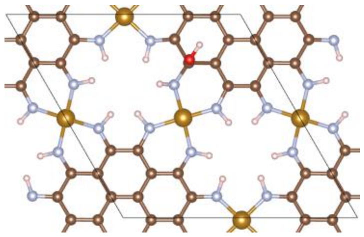	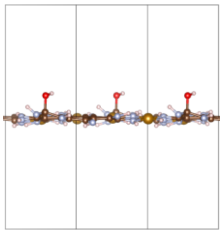
Fe-NH-MOF	OH/C2	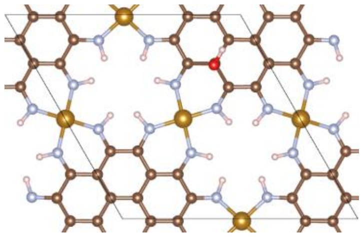	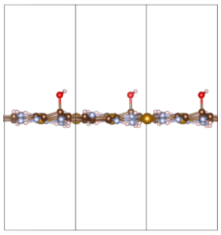
Fe-NH-MOF	OH/C3	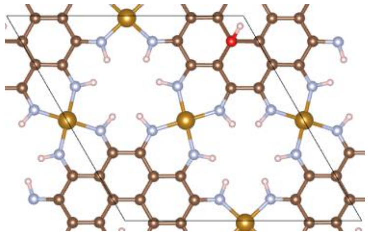	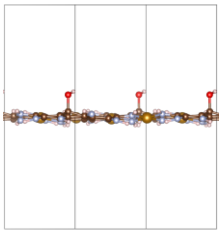
Fe-NH-MOF	O/Fe	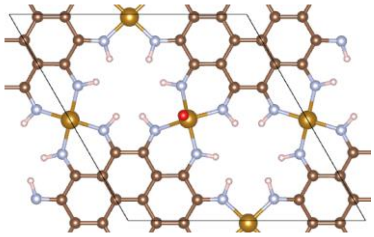	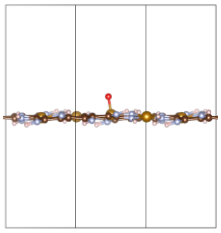
Fe-NH-MOF	O/N	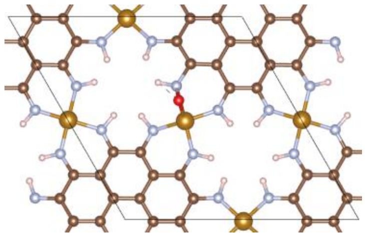	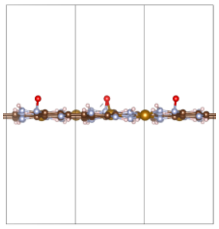
Fe-NH-MOF	O/C1	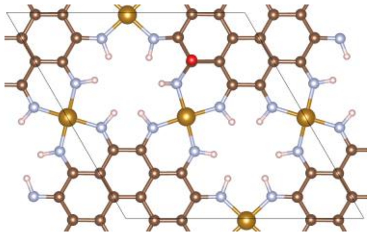	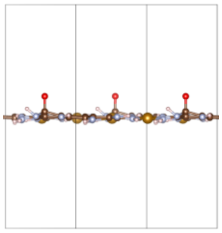

Figure S2. The optimized top and side views of H, OOH, O, and OH on Fe-NH MOF monolayer.

Materials	Adsorbate/ Site	Top view	Side view
Fe-O-MOF	-	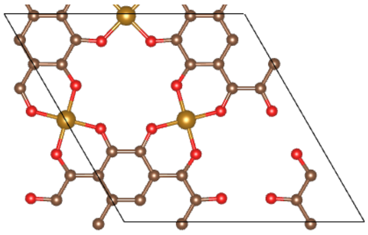	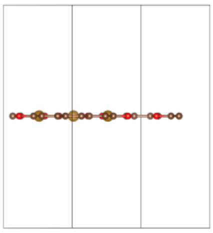
Fe-O-MOF	H/ Fe	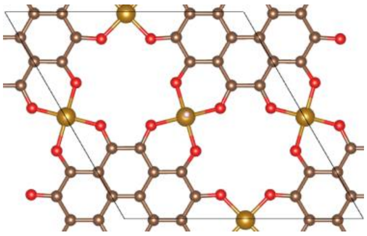	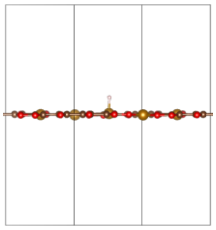
Fe-O-MOF	H/ O	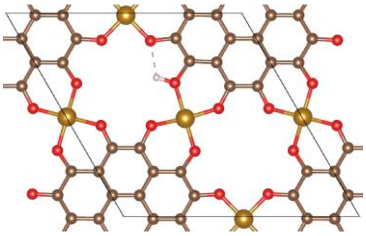	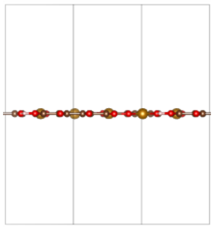
Fe-O-MOF	H/C1	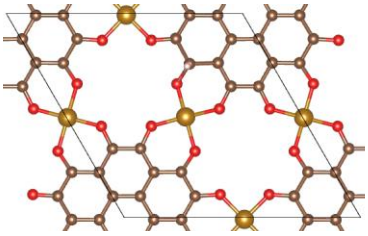	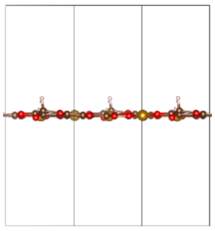
Fe-O-MOF	H/C2	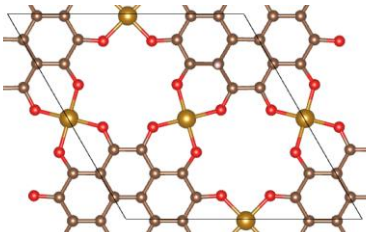	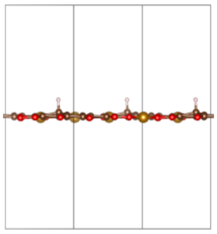
Fe-O-MOF	H/C3	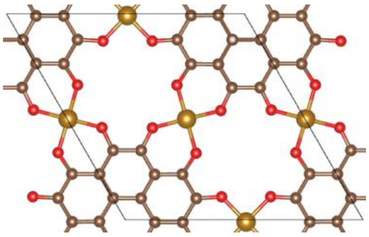	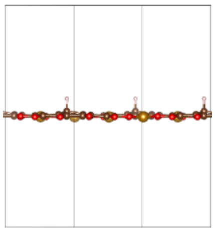

Fe-O-MOF	OOH/Fe	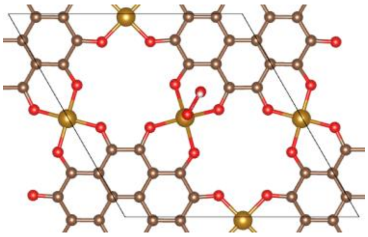	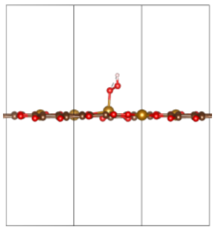
Fe-O-MOF	OOH/O	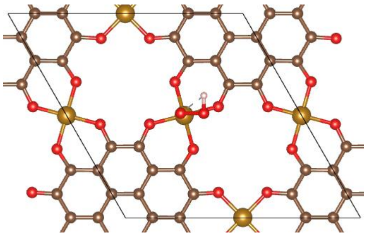	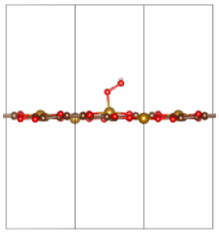
Fe-O-MOF	OOH/C1	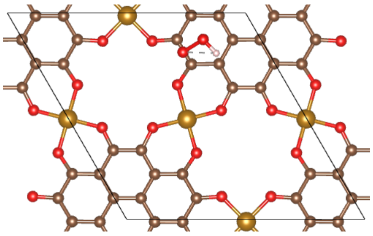	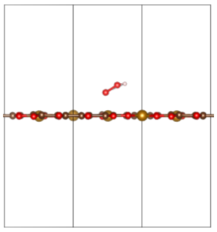
Fe-O-MOF	OOH/C2	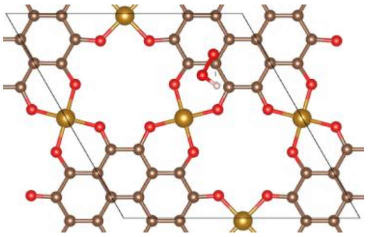	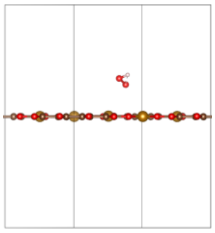
Fe-O-MOF	OOH/C3	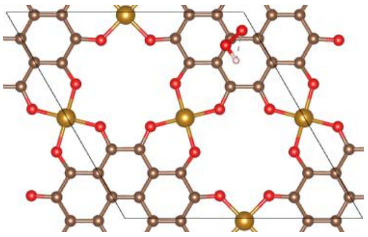	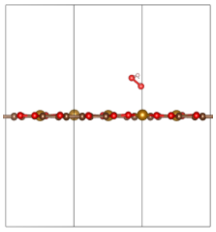
Fe-O-MOF	OH/Fe	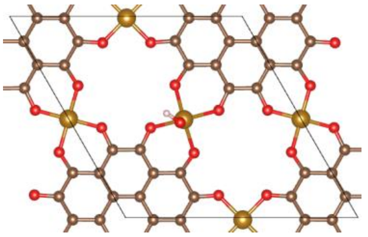	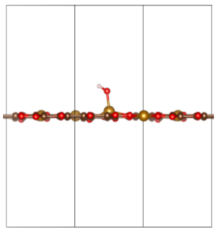
Fe-O-MOF	OH/O	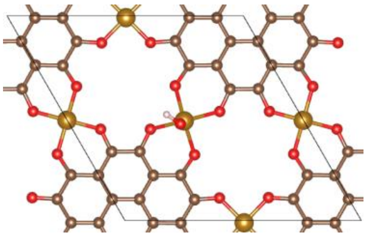	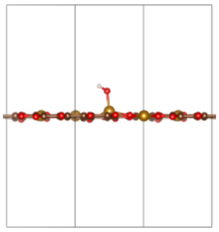

Fe-O-MOF	OH/C1	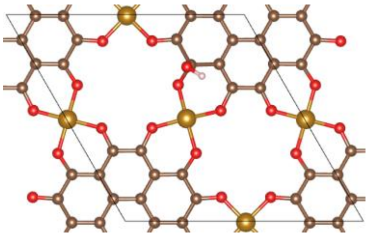	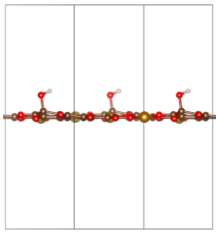
Fe-O-MOF	OH/C2	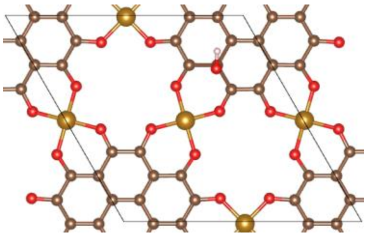	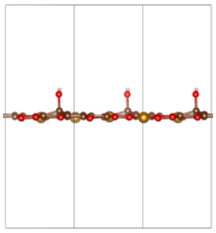
Fe-O-MOF	OH/C3	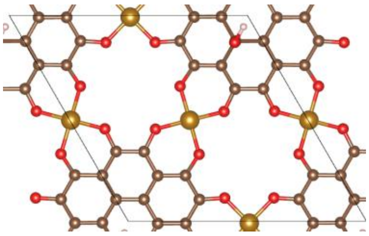	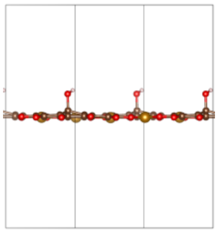
Fe-O-MOF	O/Fe	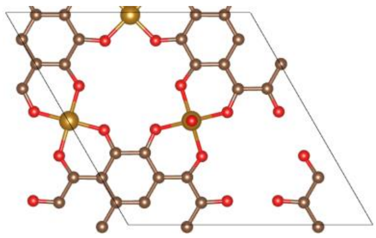	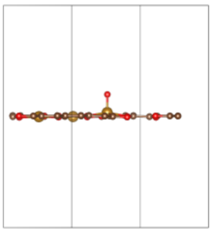
Fe-O-MOF	O/O	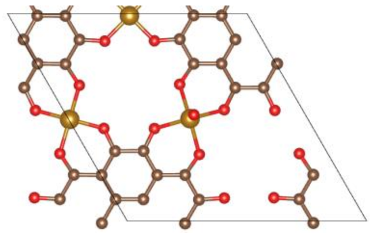	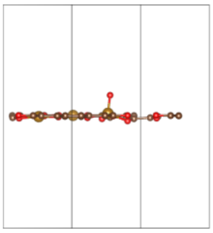

Figure S3. The optimized top and side views of H, OOH, O, and OH on Fe-O MOF monolayer.

Materials	Adsorbate/ Site	Top view	Side view
Fe-S-MOF	-	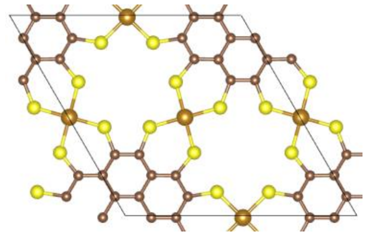	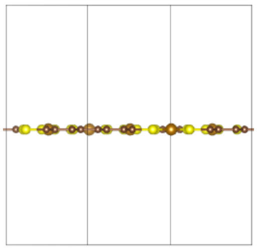
Fe-S-MOF	H/ Fe	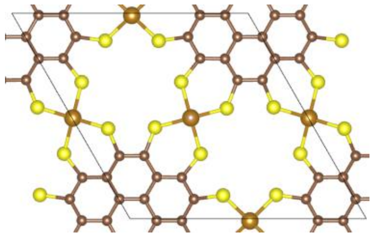	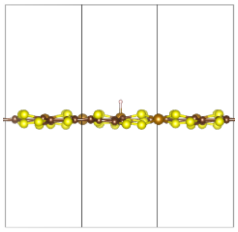
Fe-S-MOF	H/ S	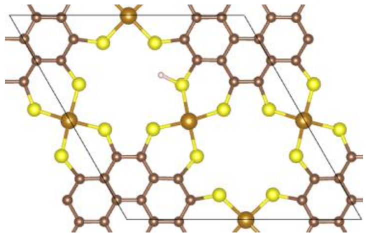	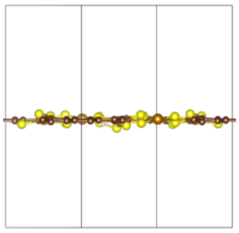
Fe-S-MOF	H/C1	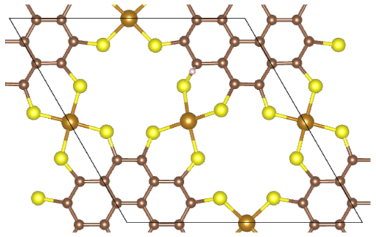	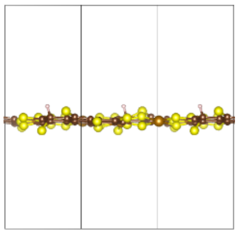
Fe-S-MOF	H/C2	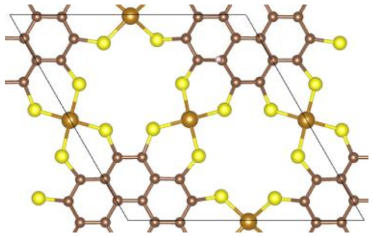	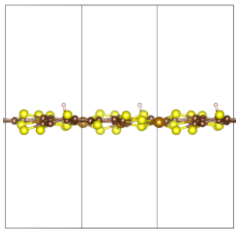
Fe-S-MOF	H/C3	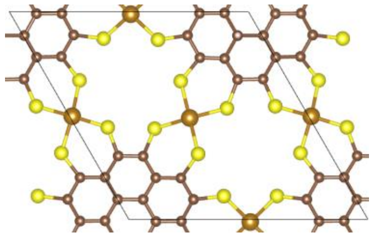	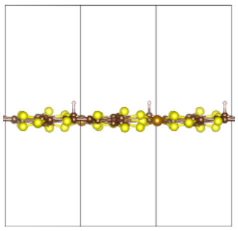

Fe-S-MOF	OOH/Fe	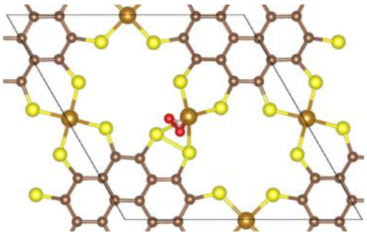	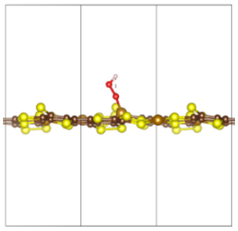
Fe-S-MOF	OOH/S	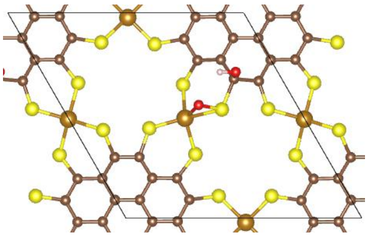	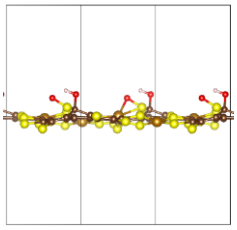
Fe-S-MOF	OOH/C1	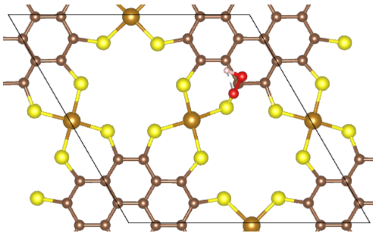	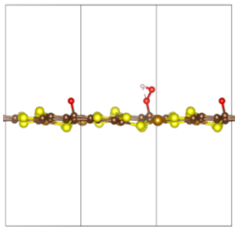
Fe-S-MOF	OOH/C2	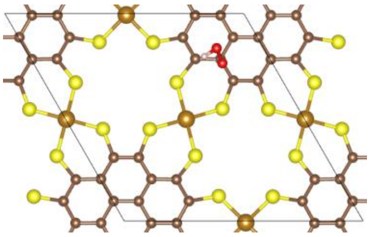	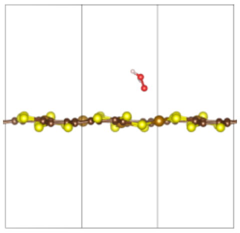
Fe-S-MOF	OOH/C3	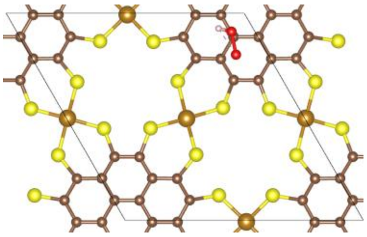	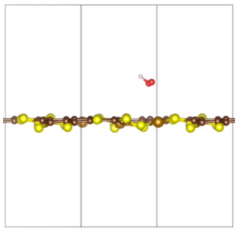
Fe-S-MOF	OH/Fe	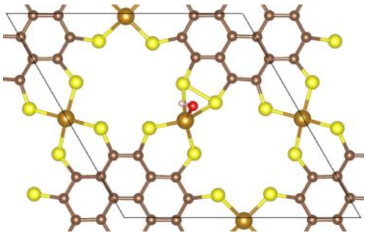	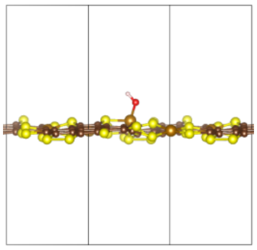
Fe-S-MOF	OH/S	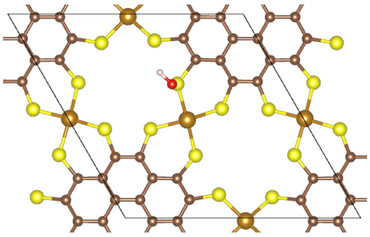	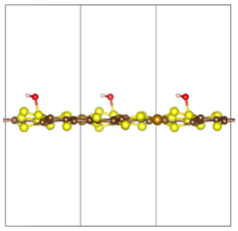

Fe-S-MOF	OH/C1	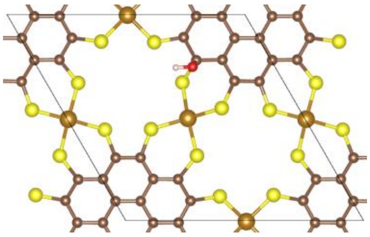	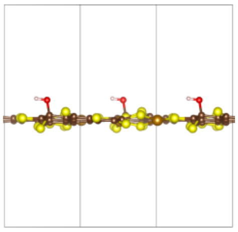
Fe-S-MOF	OH/C2	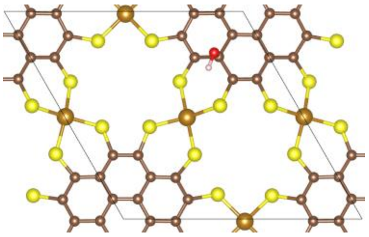	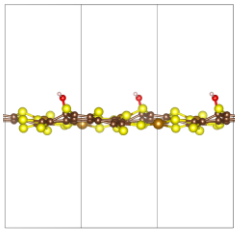
Fe-S-MOF	OH/C3	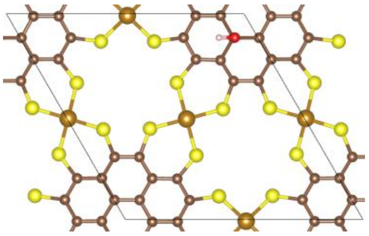	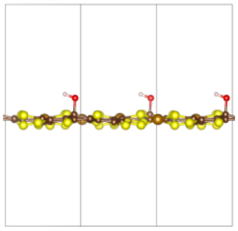
Fe-S-MOF	O/Fe	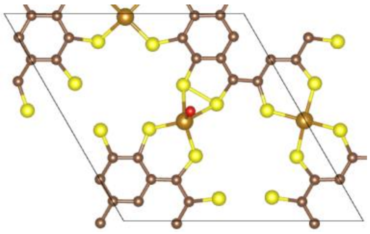	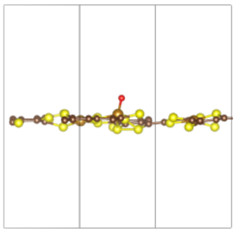
Fe-S-MOF	O/S	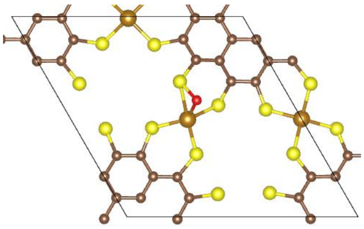	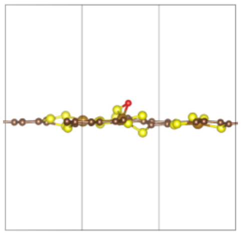

Figure S4. The optimized top and side views of H, OOH, O, and OH on Fe-S MOF monolayer.

Supplementary Tables

Table S1 Optimized lattice constants (l_a) vs total energies (E_{tot}) of Fe-NH-MOF, Fe-O-MOF, and Fe-S-MOF monolayers.

Fe-NH MOF		Fe-O MOF		Fe-S-MOF	
(l_a) (Å)	(E_{tot}) (eV)	(l_a) (Å)	(E_{tot}) (eV)	(l_a) (Å)	(E_{tot}) (eV)
12.40	-386.05408	12.00	-321.59756	12.00	-264.38453
12.50	-386.40396	12.20	-322.49340	12.28	-267.22506
12.55	-386.49375	12.25	-322.58313	13.20	-285.73598
12.58	-386.52149	12.26	-322.59447	13.55	-287.37690
12.59	-386.52601	12.27	-322.60345	13.57	-287.40530
12.60	-386.52834	12.28	-322.6090	13.60	-287.43616
**12.61**	**-386.52848**	12.29	-322.61517	13.62	-287.44920
12.62	-386.52667	12.30	-322.61840	13.63	-287.45322
12.64	-386.51676	**12.31**	**-322.61878**	13.64	-287.45576
12.66	-386.49899	12.32	-322.61743	**13.65**	**-287.45689**
12.68	-386.47318	12.33	-322.61401	13.66	-287.45649
12.70	-386.43955	12.34	-322.60868	13.67	-287.45466
12.80	-386.16279	12.35	-322.60124	13.68	-287.45121
-	-	12.40	-322.53530	13.70	-287.44023
-	-	12.50	-322.30643	13.75	-287.38960
-	-	-	-	13.80	-287.30549
